# Dry Eyes among Visual Display Terminal Users Visiting the Outpatient Department of Ophthalmology of a Tertiary Care Centre

**DOI:** 10.31729/jnma.8307

**Published:** 2023-10-31

**Authors:** Dikchhya Sharma, Sabina Shrestha

**Affiliations:** 1 Department of Ophthalmology, Kathmandu Medical College and Teaching Hospital, Sinamangal, Kathmandu, Nepal

**Keywords:** *dry eyes*, *health*, *prevalence*

## Abstract

**Introduction:**

Visual display terminal usage has increased substantially in recent years across all age groups and is considered one of the major risk factors for dry eye disease. Early assesment of dry eyes and prevention is important. The aim of this study is to find the prevalence of dry eyes among visual display terminal users visiting the Outpatient Department of Ophthalmology of a tertiary care centre.

**Methods:**

A descriptive cross-sectional study was conducted among patients visiting the Department of Ophthalmology in a tertiary care centre after receiving ethical approval from the Institutional Review Committee. Data was collected from 1 October 2021 to 31 March 2022. Convenience sampling method was used. The point estimate was calculated at a 90% Confidence Interval.

**Results:**

Among 94 visual display terminal users, the prevalence of dry eyes was 55 (58.51%) (50.1866.84, 90% Confidence Interval).

**Conclusions:**

The prevalence of dry eyes among visual display terminal users visiting the Outpatient Department of Ophthalmology was similar to other studies done in similar settings.

## INTRODUCTION

Visual display terminals (VDT) have now spread their presence from fixed desktops of office space to laptops.^1^ Visual display terminal workers are defined as employees whose primary job, for at least 20 hours per week, involves work at a visual/video display terminal (VDT).^2^ VDT use is a remarkable risk factor for dry eye symptoms.

The prolonged use of a VDT causes not only evaporated tears but also slows the blink rate and causes a malfunction of meibomian glands inducing dry eye.^3^ Dry eye symptoms also have been reported to have a negative impact on psychological health and overall well-being.^4^ Apart from tear film break-up time (TBUT), Schirmer test and ocular surface staining, there are different questionnaires, which can be used to assess dry eyes. One of them is the Ocular Surface Disease Index (OSDI) Questionnaire.^5^ Early assessment of symptoms, diagnosis and effective treatment strategies for dry eye disease is necessary to improve physical well-being and workplace productivity in patients using VDT.^6^

The aim of this study was to find the prevalence of dry eyes among visual display terminal users visiting the Outpatient Department of Ophthalmology of a tertiary care centre.

## METHODS

This descriptive cross-sectional study was conducted among patients using VDT visiting the Outpatient Department of Ophthalmology at Kathmandu Medical College Teaching Hospital, Sinamangal, Kathmandu, Nepal. Data was collected from 1 October 2021 to 31 March 2022. Ethical approval was taken from the Institutional Review Committee (Reference number: 0609202102). All the patients visiting the outpatient department during the study period with complete records were included. Patients who had undergone any ocular surgery in the preceding 6 months, or presented with systemic or ocular conditions (such as rheumatoid arthritis, Sjogren Syndrome, rosacea, infectious disease, or previous glaucoma diagnosis) that could interfere with ocular surface status were excluded. Convenience sampling method was used. The sample size was calculated using the following formula:


$$\begin{array}{ll} \text{n} & ={\text{Z}}^{2}\times \frac{\text{p}\times \text{q}}{{\text{e}}^{\text{2}}}\\ & =1.64^{2}\times \frac{0.50\times 0.50}{0.10^{2}}\\ & =68 \end{array}$$

Where,

n = minimum required sample size
Z = 1.64 at 90% Confidence Interval (CI)
p = prevalence taken as 50% for maximum sample size calculation
q = 1-p
e = margin of error, 10%

The minimum required sample size was 68. However, the final sample size taken was 94.

First, the OSDI questionnaire was administered to the participants. It is a questionnaire which includes 12 questions related to symptoms, environmental conditions which can cause dry eye and functional limitations. Each question has 5 likert type response options. 0 (none of the time) to 4 (all of the time). The total OSDI score was calculated by the formula given below.

OSDI = Sum of scores × 25/total no of questions answered.

Scores range from 0 to 100.

A cut-off value of ≥13 was taken as a diagnosis of dry eyes.^8^

0-12 representing normal,

13-32 representing mild to moderate dry eyes

≥33 representing severe dry eyes.

A complete ophthalmological examination was done which included best-corrected visual acuity, anterior and posterior segment evaluation along with tear film break up time and Schirmer test.

Data were entered in Microsoft Excel 2010 and analyzed using IBM SPSS Statistics Version 20. The point estimate at a 90% CI was calculated.

## RESULTS

Among 94 VDT users, 55 (58.51%) (50.18-66.84, 90% CI) had dry eyes. The mean age of the participants with dry eyes was 29±5.30 years. A total of 21 (38.18%) were female and 34 (61.82%) were male. Most of the participants with dry eyes, 31 (56.36%) belonged to the age group of 25-34 years. A total of 29 (52.73%) used laptops only, 8 (14.55%) used desktops only and 18 (32.73%) used both laptops and desktops. The average hours of VDT use among patients with dry eyes were 8.1±2.3 hrs. A total of 34 (61.82%) used the devices for 6-8 hours per day (Table 1).

**Table 1:** Distribution of hours of use of VDT users with dry eyes (n = 55).

VDT use (in hrs/day)	n (%)
4-6	9 (16.36)
6-8	34 (61.82)
>8	12 (21.82)

Among them, 37 (67.27%) had mild to moderate dry eyes whereas 18 (32.73%) had severe dry eyes according to OSDI (Table 2).

**Table 2:** Distribution of severity of dry eyes (n= 55).

Severity	n (%)
Mild-moderate	37 (67.27)
Severe	18 (32.73)

The Schirmers test done without topical anaesthesia showed a dry eye in only 8 (14.54%). TUBT showed values less than 10 minutes in 23 (41.81%).

## DISCUSSION

The prevalence of dry eyes was 58.51% using the OSDI questionnaire. This prevalence is lower than the study done in Mexico in similar settings.^7^ However, a study done in similar settings in Italy showed dry eyes in 50% of the participants.^9^ Literature about the prevalence of dry eyes in VDT users refers to the Asiatic population, from two large studies. The Osaka study and the Moriguchi study have reported lower values than our study.^6,10^ These contrast values can be explained by different diagnostic criteria used to diagnose dry eyes. Higher values are derived from studies in which a less restrictive definition was used and the lower are derived from those studies in which a more restrictive definition was used. The observed prevalence of dry eyes with VDT use in our study could be with the use of only OSDI as the basis for diagnosis of dry eyes.

The mean age of the patients with dry eyes was 29±5.30 years and most of them 31 (56.36%) belonged to the age group of 25-34 years. This finding was similar to the study done among Japanese VDT workers and attributed to the increasing use of computers among younger age groups.^3^ Male preponderance with 61.82% was found in our study which is in contrast to the Japanese study that has found maximum dry eyes in female patients.^3^ The average hours of VDT use among patients with dry eyes were 8.1±2.3 hrs in our study. Most of the participants in our study used VDT for 6-8 hrs/day. The increased prevalence of dry eyes in our study might be because of the inclusion of participants using VDT for >4 hrs/day. Longer durations of VDT work were observed to be associated with a significant trend toward a higher prevalence of dry eye symptoms.^3^

The pathophysiological mechanism for dry eyes may be decreased blink frequency and increased incomplete blinking with VDT use. This reduces the secretion and distribution of meibum to the lipid layer of the tear film on the ocular surface, lowering the stability of the tear film. which finally causes a shorter TBUT. Tear film break-up time showed values less than 10 secs in 23(41.81%) patients in our study similar to the study done in similar settings in Italy (43.3%).^9^ However, the Schirmer test showed dry eyes in only 14.54% of participants similar to the Osaka Study.^3^ Most of the participants had a normal lacrimal function. This finding differs from the study done in Japan among 1025 office workers which suggested that the cumulative years of VDT use might result in aqueous deficiency.^10,11^

With increasing evidence of the deleterious effect of continuous VDT usage, the obvious solution, although often impractical, would be to limit prolonged screen time. Optimizing VDT positioning, lifestyle modifications, blinking exercises, use of artificial tear drops and workstation humidifiers might serve as further ancillary treatments.^12^

The limitation of this study was that this was a singlecentred study and the sample size was small. So, this cannot be generalised to the whole population. We have determined the prevalence using only OSDI. Further large-scale studies need to be done to define dry eyes and determine prevalence according to the standardized criteria using symptoms combined with TBUT, tear film osmolarity and ocular surface staining.

## CONCLUSIONS

The prevalence of dry eyes among VDT users visiting the Outpatient Department of Ophthalmology was similar to other studies done in similar settings.
